# Theory of Mind in Pre-school Aged Children: Influence of Maternal Depression and Infants’ Self-Comforting Behavior

**DOI:** 10.3389/fpsyg.2021.741786

**Published:** 2021-11-24

**Authors:** Nora Nonnenmacher, Mitho Müller, Joana Taczkowski, Anna-Lena Zietlow, Beate Sodian, Corinna Reck

**Affiliations:** ^1^Center for Psychosocial Medicine, Heidelberg University Hospital, Institute of Medical Psychology, Heidelberg, Germany; ^2^Department of Psychology, Ludwig Maximilians University Munich, Munich, Germany; ^3^Department of Child and Adolescent Psychiatry, Psychosomatics and Psychotherapy, University Medical Center Hamburg-Eppendorf, Hamburg, Germany; ^4^School of Social Sciences, University of Mannheim, Mannheim, Germany

**Keywords:** theory of mind, maternal depression, infant self-comforting behaviors, Face-to-Face Still-Face paradigm, gender differences in infancy

## Abstract

A milestone of child development is theory of mind (ToM): the ability to attribute mental states, especially beliefs and desires, to other persons and to understand that their behavior is guided by mental states. The learning process about the mental world also takes place in social communication and interaction, beginning in infancy. Infancy is assumed to be a sensitive period for the development of social skills through interaction. Due to limited self-regulatory skills, infants depend on sensitive behavior of their caregivers to regulate affective states and physiological arousal, and in turn, mutually regulated affects allow the infant to gradually acquire the capability to self-regulate negative affective states. Effective and adequate affect regulation is an important prerequisite for environmental interaction and thus for the development of socio-emotional skills. The present study investigated the relation of self-regulatory abilities in infancy and later ToM in pre-school aged children of clinically depressed mothers and healthy controls. The sample comprised of *N* = 55 mother–child dyads, *n* = 22 diagnosed with postpartum or lifetime depression according to DSM-IV and *n* = 33 healthy controls. Mother–infant-interaction was videotaped during the Face-to-Face Still-Face paradigm. At 3 and 42 months postpartum mothers were interviewed with the Structured Clinical Interview for DSM-IV Axis I Disorders (SCID-I) to evaluate maternal psychopathological status according to DSM-IV. At the age of *M* = 4.0 years, children’s ToM abilities were assessed using content-false-belief and location-false-belief tasks. The results of this study show that contrary to our hypotheses, maternal depression did not impair the development of children’s ToM-abilities *per se*. Rather, an interaction effect highlights the role of infant’s self-comforting behavior during mother–infant interaction in infancy (3 months postpartum) for ToM-development at pre-school age assessed with the Maxi-task; this association was distinct for female in comparison to male children. The results of this longitudinal study shed light on the discussion, how maternal depression influences child development and point in the direction that self-comforting behaviors in infancy can also be seen as a resource.

## Introduction

An essential domain of children’s cognitive development is the ability to attribute mental states, especially beliefs and desires, to other persons and to understand that their behavior is guided by such mental states (Premack and Woodruff, 1978). This so-called theory of mind (ToM) develops between 3 and 5 years of age (Wellman et al., 2001). Carpendale and Lewis (2004) argue that children develop social cognition (knowledge of the world as well as knowledge of other people) through triadic interaction and the child’s environment: learning about the mental world takes place during social communication and interaction, the caregiver functions as a social partner presenting mental state concepts, whereby the child gradually develops an understanding about the inner world and acquires the knowledge that individuals can have beliefs that differ from one to another.

Therefore, infancy is assumed to be a sensitive period for the development of social skills. One variable of special importance for child development is self-regulation: the ability of the child to manage distress and negative states and to maintain positive states (Kopp, 1989). As Beeghly and Tronick (2011) argue: “Such distress continues at high energetic cost, precluding the child’s engagement with the world and if chronic, compromising development.” Thus, the social development might be impaired by compromised regulatory capacities. As infants only have a limited repertoire of self-regulatory behaviors during the first months of life, such as hand-to-mouth movements and non-nutritive sucking, caregivers are thought to play an important role in the development of self- and affect regulation in infants. If caregivers interact in a supportive way, their infants benefit from dyadic processes, such as affect regulation and interactive repair (Feldman, 2007; Reck et al., 2011; Müller et al., 2015; Noe et al., 2015; Zietlow et al., 2019). Previous research indicated, that infants show increased self-comforting behaviors if caregivers are unable to co-regulate infant’s affect (e.g., due to psychiatric disorders) (Granat et al., 2017). In this case, increased self-comforting behavior in infancy was seen as an indicator for an increased developmental risk due to an inadequate affective response of the caregiver and as an indicator that child’s emotion and interpersonal regulation fails (Gianino and Tronick, 1988; Tronick and Cohn, 1989; Müller et al., 2016; Granat et al., 2017). Nevertheless, the ability to regulate affects *via* self-comforting behavior could also be seen as a resource. Cole et al. (2004) discuss, that infants first have basic self-regulatory capacities, then engage with caregivers for external stress regulation, and finally develop an array of additional self-regulatory strategies over the toddler and preschool years. With regard to children’s ToM, Fonagy et al. (2002) assume, that it can only develop on the basis of adequate affect regulation competencies.

Besides affect regulation, maternal depression may add to a specific risk constellation for ToM development, as maternal depression can be seen as a risk factor for adequate dyadic regulation. Depression belongs to the most common psychiatric disorders and often affects women during the peripartum period with prevalence rates up to 11.9% (Woody et al., 2017). For Germany, prevalence rates of 6% for postpartum depression are reported (Reck et al., 2008). Findings highlight the negative influence of maternal depression on dyadic affect and stress regulation (Reck et al., 2004) and corroborate the hypothesis, that children of depressed mothers have difficulties to develop adequate stress- and affect-regulation skills, what might affect child development in the long term (Reck et al., 2016). One factor accounting for this risk might be maladaptive interactional patterns between a depressed mother and her child, which might in turn affect the development of social skills in children (Coyl et al., 2002; Field et al., 2006; Lindsey et al., 2013; Danzig et al., 2015; Reck et al., 2016; Fadda and Lucarelli, 2017). Thus, one might assume a general mediatory role of maternal caregiving regarding the associations between maternal disorder and infant development. Nevertheless, there are also studies that did not find differences in the interactional characteristics in depressed compared to healthy mothers (Carter et al., 2001; Weinberg et al., 2008; Sidor et al., 2011). Therefore, we cannot generally assume that the quality of mother–infant-interaction is impaired in dyads, in which mothers suffer from depression. In fact, there are also studies suggesting a moderating role of maternal caregiving regarding the discussed associations (Kaplan et al., 2008; Grant et al., 2010a,b). Even though these studies demonstrated the moderation by maternal caregiving for prepartum disorders and discuss the results in the light of fetal programming, we suggest applying the idea of a moderative risk constellation between disorder and caregiving to the postpartum period. When regarding infant self-comforting behavior, the results of Müller et al. (2016) suggest that there is not a general mediation path between maternal disorder and infant self-regulation, whereas maternal bonding, a construct conceptually and empirically close to maternal caregiving (Noorlander et al., 2008), was investigated as a mediator in an sample of postpartum anxious mothers.

Moreover, negative effects of maternal depression on child development were reported, e.g., for socio-emotional (Madigan et al., 2018; Barnes and Theule, 2019) and physical development (Pierce et al., 2020). A few studies investigated the association between maternal depression and ToM development and reported a direct negative effect of maternal depression on children’s ToM performance at 4 and 5 years (Rohrer et al., 2011; Shamblaw et al., 2019). However, another study did not find impairments in the ToM of children of depressed mothers (Greig and Howe, 2001). To date, the exact mechanisms and pathways of ToM development in children of depressed mothers are not yet entirely clarified.

Besides maternal depression, child gender is also discussed as an important variable in the consideration of specific risk constellations for child development. Recent research suggests a different use of self-directed regulation strategies for male and female infants (Müller et al., 2016). Furthermore, male infants show greater difficulties than female infants in maintaining interactive regulation, and mother–son dyads take longer to repair interactive errors (Weinberg et al., 1999). Gender differences seem also evident when considering the effects of maternal depression, as mother–son dyads have a less well-adapted interaction than mother–daughter dyads with highly depressive mothers (Weinberg et al., 2006) and maternal depression is only significantly associated with cognitive outcomes in boys (Ahun et al., 2021). Moreover, male infants may be more sensitive to distress due to differences in hormonal function and cortisol responses (Elsmén et al., 2004).

In sum, the development of ToM can be impaired in children of depressed mothers and an association to the child’s affect regulation is discussed. To the best of the author’s knowledge, this is the first study focusing on specific risk constellations for ToM development, investigating possible associations between infant self-comforting behavior during interaction with their mothers and later ToM-development in the light of maternal depression.

We hypothesize that maternal depression impairs offspring’s development of a ToM at pre-school age. So far, there are studied indicating a direct association between maternal depression and child outcome, while other studies do not find a direct association. Therefore, the exact circumstances, under which maternal depression exerts influence on child development, are not yet entirely clarified. This study uses a moderation analysis to approach this research question. We hypothesize that the association between maternal depression and child development is moderated by self-comforting behavior during mother–child interaction in the early postpartum period. A moderation examines the combined effect of two variables, the predictor variable (maternal depression) and the moderator (infant self-comforting), and analyzes how the relationship between the predictor and the outcome variable changes as a function of the moderator variable (Field, 2013). Therefore, moderation analysis is of use when examining when or under what circumstances a predictor exerts influence on the outcome variable. To date, there are no studies exploring infant self-comforting behavior as a possible moderator between maternal depression (predictor variable) and ToM-development of children at pre-school age (outcome variable). We hypothesize, that the relationship between maternal depression and child development increases as a function of how distinct infant self-comforting behavior is, as self-comforting behavior can be seen either as a resource or as an indicator for an increased developmental risk due to an inadequate affective response of the caregiver. Besides infant self-comforting behavior, we expect the associations between maternal depression and child development to be influenced by infant gender; however, to the best of the author’s knowledge, the direction of effects cannot be derived by literature. Thus, we investigate the moderation effects of infant gender exploratory. To investigate whether the relationship between maternal depression and child development is much more mediated (rather than moderated), we conducted an exploratory analysis of child self-comforting behavior as a mediator. In this study, postpartum and lifetime depression were focused, as a remission of psychiatric symptoms does not necessarily lead to an improvement of the quality of the mother–child relationship and interaction (Thomas et al., 2015; Nonnenmacher et al., 2016), Therefore, not only acute depressive episodes but also remitted and lifetime diagnoses may exert negative long-term influence on mother–child relationship and interaction.

## Materials and Methods

### Procedure and Participants

Participating mothers (*N* = 108) gave birth between May 2009 and December 2012 and were recruited in local maternity hospitals (*n* = 104) and the mother–infant unit (*n* = 4) of the Psychiatric University Hospital Heidelberg. Eligible mothers were screened for depressive syndromes according to DSM-IV, and subsequently interviewed with the Structured Clinical Interview for DSM-IV Axis I Disorders (SCID-I) (Wittchen et al., 1997) within the first 3 months postpartum (TI). At this first assessment point, the Face-to-Face Still-Face paradigm (FFSF; Tronick et al., 1978) was videotaped. The FFSF consists of three episodes each lasting 2 min: first, an initial face-to-face interaction in which the mothers are instructed to play with their infant as usual (without the aid of toys and pacifiers). Next, the still-face episode in which the mothers have to turn their head aside while silently counting to 10 and then turn back to the infant but not engage in any gestures, facial expressions, or vocalizations. Finally, the procedure ends with the reunion episode in which the mother is required to resume face-to-face play with her infant.

Four years postpartum (TII), mother–child dyads were re-invited to the lab. At this follow-up assessment, children’s ToM was assessed with a location and a content false belief task.

Regarding the first assessment point 3 months postpartum, the initial sample size of *N* = 108 mothers was reduced to *n* = 92 due to the exclusion of women without depression but with other psychiatric diagnoses (*n* = 11) and missing FFSF (*n* = 5). Four years postpartum, sample size was reduced to *n* = 55 due to missing follow-up data. Women were assigned to the healthy (*n* = 33) or depressed (*n* = 22) group according to their mental health status postpartum (TI). The healthy group consisted of mothers without current or lifetime psychiatric disorders. Characteristics of the depressed group are reported elsewhere (Nonnenmacher et al., 2016).

All participating mothers needed to be at least 18 years old and have adequate knowledge of the German language. Exclusion criteria for children were prematurity (born before the completion of the 36th week of gestation), small-for-gestational-age or congenital abnormalities. None of the children met these exclusion criteria.

The independent Ethics Committee of the Heidelberg Medical Faculty had previously approved the study protocol. Written informed consent was obtained after study procedures had been fully explained.

### Sample Characteristics

Maternal age at birth ranged from 24 to 43 years with a mean of *M* = 33.40 years (SD = 4.1) within the overall sample (*n* = 55).

Infant’s age at TI ranged from 12.7 to 17.9 weeks with a mean age of *M* = 14.6 (SD = 1.4) weeks. At pre-school age (TII), child’s age ranged from 3.1 to 4.2 years with a mean age of *M* = 4.0 (SD = 0.2) years. With regard to the total sample, *n* = 23 (41.8%) children were female and *n* = 32 (58.2%) were male. The distribution of maternal education level in the overall sample was as follows: *n* = 13 (16.4%) completed intermediate secondary education, *n* = 9 (23.6%) qualified for university admission, and *n* = 33 (60.0%) held a university degree. Detailed sociodemographic information are displayed in Table 1.

**TABLE 1:** Maternal and child demographics and tests on comparability of subgroups.

	General	Control	Clinical	*t* (*p*)		General	Control	Clinical	*t* (*p*)
Infant age at T1 (weeks)^a^	14.6 (1.4)	14.6 (1.5)	14.5 (1.2)	0.35 (0.72)	Child age at TII (years)^b^	4.0 (0.2)	4.0 (0.2)	4.0 (0.1)	−0.67 (0.51)
*M* (SD)					*M* (SD)				
Child IQ at TII (IQ norm)^c^	97.7 (10.6)	96.8 (10.7)	99.3 (10.4)	−0.85 (0.40)	Maternal age at birth (years)^d^	33.4 (4.1)	33.0 (3.9)	34.2 (4.3)	−1.08 (0.28)
*M* (SD)					*M* (SD)				

**Child gender (frequencies)**	**General**	**Control**	**Clinical**	**χ^2^ (*p*)**	**Bilingualism at TII (frequencies)**	**General**	**Control**	**Clinical**	**χ^2^ (*p*)**

Female infants	23	14	9	0.01^e^ (0.91)	False	41	26	15	0.24^f^ (0.88)
Male infants	32	19	13		True	6	4	2	

**Maternal education at T1 (frequencies)**	**General**	**Control**	**Clinical**	***U* (*p*)**	**Number of children at T1 (frequencies)**	**General**	**Control**	**Clinical**	***U* (*p*)**

University degree	33	21	12	308.5 (0.29)	1 child	32	20	12	358.0 (0.92)
University entrance qualification	9	7	2		2 children	15	7	8	
Secondary qualification	13	5	8		3 children	8	6	2	

**Partnership at T1 (frequencies)**	**General**	**Control**	**Clinical**	**χ^2^ (*p*)**	**Occupation at T1 (frequencies)**	**General**	**Control**	**Clinical**	**χ^2^ (*p*)**

False	2	0	2	3.11^g^ (0.08)	False	53	32	21	0.09^h^ (0.77)
True	53	33	20		True	2	1	1	

*^a^min = 12.7; max = 17.9;*

*^b^min = 3.1; max = 4.2;*

*^c^min = 71.0; max = 124.0;*

*^d^min = 24.1; max = 43.1;*

*^e^0 cells have expected count less than 5, minimum expected count is 9.2;*

*^f^2 cells have expected count less than 5, minimum expected count is 2.2;*

*^g^2 cells have expected count less than 5, minimum expected count is 0.8;*

*^h^2 cells have expected count less than 5, minimum expected count is 0.3.*

### Measures

#### Structured Clinical Interview for DSM-IV Axis I Disorders

The German version of the SCID-I (Wittchen et al., 1997) is a semi-structured and a widely used interview for the diagnosis of selected Axis I disorders. It was used by trained and experienced clinical psychologists for the assessment of maternal depression.

#### Coding of Infant Behavior During the Face-to-Face Still-Face: Infant and Caregiver Engagement Phases-Revision

As described in Müller et al. (2016), the FFSF was videotaped and coded by two trained and reliable coders using the German translation and revision of the micro-analytical Infant and Caregiver Engagement Phases-Revision (ICEP-R; Reck et al., 2009), using the Noldus Observer Video-Pro ^®^ coding system with 1 s time intervals. The coders were blind to the hypotheses of the study and the maternal psychiatric status.

The ICEP-R combines information from the infant’s and caregiver’s face, direction of gaze and vocalizations. Additionally for infants and co-occurring to the maternal and infant engagement codes (as described, e.g., Müller et al., 2015), oral and manual self-comforting behaviors were coded. Oral self-comforting (ISCO) included: (1) the infants’ initiated skin contact between their own body parts and their mouth, (2) the infants’ initiated mouth contact to exogenous objects, or (3) sucking on the caregiver’s hand or fingers (self-initiated or not). Manual self-comforting behaviors (ISCH) are coded if the infants touch one hand with the other. The primary independent measures were the sum of relative time proportions of infant self-comforting behaviors. In the following analyses, the relative time proportions for ISCO and ISCH (sum of seconds in which infants engage in oral or manual self-comforting behaviors divided by the time of the FFSF) were added. As both codes may occur simultaneously, the sum of sores can add up to >1 or to >100%, as the descriptive results of single codes are multiplied with 100%. In mean, infants engaged 11.54% of interaction time in self-comforting behaviors (SD = 2.27%).

#### Theory of Mind Abilities at Pre-school Age

Two false belief tasks were used to assess ToM reasoning, one assessing children’s understanding of a false belief about the content of a container, and one assessing their understanding of a false belief about the location of an object.

#### Content False Belief Tasks

Content false belief tasks (Perner et al., 1987; Gopnik and Astington, 1988) measure if children are able to distinguish between two different beliefs concerning one state of affairs and if they are able to represent them simultaneously.

The experimenter shows the children a familiar container (a “Smarties” box) and asks, “What do you think is inside it?” (control question 1). Children are then shown that there is an unexpected content in the container (stones). The experimenter takes the stones out of the box, shows them to the child, saying “oh, there are stones in the box,” and then puts them back into the box, closing the lid. Subsequently children are asked, “Before we opened the box, what did you think was in here?” (test question 1). Now the children have to distinguish between their former belief (sweets inside) and their current knowledge (stones). They have to represent these two different mental concepts simultaneously. Afterward, the children are shown a puppet. They are informed that the puppet does not know anything. The children are asked: “What does the puppet think is inside the box? Smarties or stones?” (test question 2). Once more the children need to represent their actual belief (stones inside) and the puppet’s belief, even if they differ. As another control question, the children were asked, if the puppet has ever looked in the box (control question 2).

The children get one point for each correct answer to the test questions, two in sum. Child’ responses to the test questions were only counted, if the two control questions were answered correctly.

In the presented study, *n* = 17 children solved neither test question 1 nor test question 2, *n* = 21 solved one test question correctly and *n* = 15 solved both test questions. *n* = 2 children could not be assessed, thus, have missing values in the analysis regarding the Smarties-score.

#### Location False Belief Task

Second, a widely used location false belief task was assessed, the so-called Maxi-task (Wimmer and Perner, 1983). Children are told a story about Maxi, who puts chocolate in a closet and goes away. In the meantime, while Maxi is not in the room, his mother uses the chocolate for baking and then stores the chocolate in another closet and leaves the kitchen. After Maxi returned to the kitchen, children were asked: “Where will Maxi look for the chocolate?” (test question 1). The children have to represent Maxi’s false belief independently of their own knowledge of the current location of the chocolate. As a reminder question, the children were asked where the chocolate is actually located.

The story goes on, Maxi’s brother asks him for the chocolate and Maxi tells him the wrong closet so that the brother will not find the chocolate. The children were asked: “What does Maxi tell his brother where the chocolate is?” (test question 2). The children need to represent Maxi’s belief to give the correct answer even if Maxi’s belief is wrong and differs from their own belief. Therefore, test question 2 tests the understanding of the consequences of the false belief understanding (test question 1). Finally, a second reminder question and a control question were asked (“Where is the chocolate actually located?” and “Where was the chocolate stored at the beginning?”). Child’ responses to the test question were only counted, if the control question and the reminder questions were answered correctly.

The points were awarded as follows: children get zero point, if neither test question 1 nor test question 2 was answered right *or* if only test question 2, but not test question 1 was answered correctly (as this might be a coincidence and cannot be seen as evidence for false belief understanding). One point was awarded, when only test question 1, but not test question 2 was answered correctly, two points were awarded, when both test questions were answered correctly (min = 0; max = 2).

In our study, *n* = 23 children solved neither task or only test question 2 (0 points), *n* = 21 solved test question 1 (1 point), and *n* = 9 solved both tasks (2 points). *n* = 1 child could not be assessed, thus, has a missing value in the analysis regarding the Maxi-score.

#### Child’s Cognitive Development

German Version of the Wechsler Preschool and Primary Scale of Intelligence – III: Hannover–Wechsler Intelligence Test for preschool children – III (HAWIVA-III) (Ricken et al., 2007).

Child’s cognitive development was assessed with the HAWIVA-III by a trained psychologist. Children performed eight subtests (Mosaic test, General knowledge, Laying figures, Vocabulary test, Image concepts, Symbol search, Recognize terms, and Coding symbols); the full-scale intelligence quotient (IQ) was calculated from these subscales (age-standardized). IQ-scores of 85–115 (*M* = 100; SD = 15) are classified as average.

##### Demographic information form and informed consent

Each woman completed a demographic information form at both measurement points, including general data about children, bilingualism of the child, maternal educational level, and age.

### Statistical Analyses

We used the *Statistical Package for Social Sciences* (IBM™ SPSS ^®^ v. 26.0.0.0) for all the analyses conducted in this study. Power-estimations for the confirmative analysis were computed using G-Power v. 3.1.9.7 (Faul et al., 2007, 2009). Little’s (1988) missing completely at random (MCAR)-test was used to evaluate if the list-wise case-exclusions as described in the participants section were valid for our data set before carrying out the main analyses. The MCAR-test analyses if the MCAR-condition is fulfilled. If non-significant, differences between excluded cases and the remaining sample are unlikely and missing values are unlikely to depend on third variables. To ensure comparability between the groups, differences regarding demographic characteristics between controls and their clinical counterparts were explored (*via t*-tests, *U*-tests, and χ^2^-tests).

Since the distributions of most of parametric study variables were significantly skewed (*p* < 0.01 in Kolmogorov–Smirnov and Shapiro–Wilk test), we used generalized linear modeling (with robust maximum likelihood estimation), as general linear model may lack sufficient robustness against the violation of mathematical assumptions (e.g., normal distribution) and thus may lead to progressive statistical decisions (especially in small samples and between unequally sized groups). The hypotheses were tested in two models, one for each measure of child ToM abilities (location and content false belief task). As two models were estimated, the critical α-error for the main analyses was Bonferroni–Holm adjusted to α = 0.025 for the first and α = 0.05 for the second model. Empirical *p*-values are two-tailed. As estimator for effect sizes $w^{2}\,\left(\frac{\chi^{2}}{N}\right)$ was computed for significant results. According to Cohen’s (1977) conventions, *w*^2^ = 0.01 are small, *w*^2^ = 0.09 are medium-sized, and *w*^2^ = 0.25 are large effects.

Exploratory, we evaluated if infant self-comforting behaviors mediated between diagnostic group and the ToM measures using conditional process analyses *via* the SPSS ^®^ Makro “PROCESS” (v. 3.5) (Hayes, 2018). We tested a simple mediation model (model 4). The standard errors and confidence intervals of the indirect (mediated) effects are bootstrapped and bias corrected (*n* = 5,000 samples). Empirical *p*-values are two-tailed and were not adjusted due to the exploratory nature of these analyses (critical α = 0.05).

## Results

### Preliminary Analyses

In order to ensure comparability between the clinical and the control group, the distributions of demographic variables were compared using *t*-, *U*-, and χ^2^-tests. As demonstrated in Table 1 no differences were found between the groups. Analyses regarding comparability of sociodemographic variables between depressed and healthy group revealed no significant differences (see Table 1).

For the MCAR-test, we considered the following variables: socio-demographic data (e.g., age), interaction variables and matching data (ICEP-R), as well as questionnaire data (Beck Depressions Inventory, BDI-II; Hautzinger et al., 2009); German version of Child Behavior Checklist (CBCL 1,5-5; Arbeitsgruppe Deutsche Child Behavior Checklist, 2000); Partnership Questionnaire (PFB; Hahlweg, 1996); and Edinburgh Postpartum Depression Scale (EPDS; Bergant et al., 1998). The test was non-significant (χ^2^ = 3,138.76, df = 5,033, *p* = 0.99). Therefore, the list-wise case-exclusions were valid for our sample and the sub-population is representative for the larger sample.

### Main Analyses

We used generalized linear modeling with robust maximum likelihood estimations to evaluate the hypotheses in two models, one for each child ToM abilities. The dependent variables were the ToM-scores described above (Smarties- and Maxi-score). We included the diagnostic group and child gender as dummy-coded variables (index categories were the control group and female gender) as well as self-directed regulation as main effects in the model. Additionally, we included a diagnostic group × child gender, a diagnostic group × self-directed regulation as well as a child gender × self-directed regulation interaction term, to evaluate if a potential effect of self-directed regulation is moderated by group or gender.

As demonstrated in Table 2, regarding the Smarties-score there was no significant main or interaction effect (*p* > 0.08). Moreover, the omnibus test was non-significant (*p* = 0.53). The power for this analysis was approximated for linear multiple regressions. The chance of finding a large effect (*f*^2^ = 0.35) of single coefficients in our sample was had a power of 1−β = 0.99. Medium-sized effects (*f*^2^ = 0.15) could be detected with a power of 1-β = 0.79. Only small effects (*f*^2^ = 0.02) could not sufficiently be excluded with a chance of 17.2% for detection.

**TABLE 2:** Generalized linear regression model on theory of mind measures.

	Parameter	*B*	SE	Lower CI bound (95%)	Upper CI bound (95%)	Wald χ^2a^	*p*
Model 1: Smarties score (*n* = 53)^a^	Control group	–0.540	0.316	−1.159	0.078	2.931	0.087
	Female gender	–0.226	0.345	−0.901	0.450	0.428	0.513
	Self-directed regulation	–1.026	1.076	−3.136	1.083	0.909	0.340
	Group × gender	0.167	0.422	−0.660	0.995	0.157	0.692
	Group × regulation	2.161	1.326	−0.438	4.761	2.655	0.103
	Gender × regulation	0.673	1.496	−2.258	3.604	0.202	0.653
Model 2: Maxi score (*n* = 54)^b^	Control group	0.330	0.256	−0.172	0.831	1.660	0.198
	Female gender	0.172	0.305	−0.427	0.771	0.317	0.574
	Self-directed regulation	1.527	0.900	−0.237	3.290	2.879	0.090
	Group × gender	–0.424	0.358	−1.126	0.278	1.403	0.236
	Group × regulation	–1.056	0.979	−2.974	0.862	1.165	0.280
	Gender × regulation	3.564	0.923	1.755	5.372	14.919	<0.001

*^a^Likelihood-ratio omnibus test compares the fitted model against the intercept-only model, χ^2^ = 5.09, p = 0.53; intercept: B = 1.26, SE = 0.23, 95% CI = [0.81; 1.71], Wald χ^2^ = 30.47, p < 0.01.*

*^b^Likelihood-ratio omnibus test compares the fitted model against the intercept-only model, χ^2^ = 13.82, p = 0.03; intercept: B = 0.34, SE = 0.17, 95% CI = [0.00; 0.67], Wald χ^2^ = 3.90, p < 0.05.*

Regarding the Maxi-score, the omnibus test was significant (*p* = 0.03). There was no significant main effect (*p* > 0.09), however, there was a significant interaction term between child gender and self-directed regulation (*p* < 0.01), indicating a large effect (*w*^2^ = 0.28): according to this interaction effect, the association between self-directed regulation and child Maxi-score is pronounced for female in comparison to male gender. The other two interaction effects were not significant (*p* > 0.23). The power for this analysis was approximated for linear multiple regressions. The chance of finding a large effect (*f*^2^ = 0.35) of single coefficients in our sample was had a power of 1-β = 0.99. Medium-sized effects (*f*^2^ = 0.15) could be detected with a power of 1-β = 0.80. Only small effects (*f*^2^ = 0.02) could not sufficiently be excluded with a chance of 17.5% for detection.

### Additional Analyses

Although the distributions of child IQ and bilingualism at TII did not differ between the groups and thus do not fulfill a main criterion for confounding, the analyses were repeated with these potential confounders as covariates.

Still, there are no other significant main or interaction effects regarding the Smarties-score (*p* > 0.16).

Regarding the Maxi-score, the interaction effect between child gender and self-directed regulation remains significant (*p* < 0.01). Additionally in this model there are no other significant main or interaction effects (*p* > 0.21).

Furthermore, we analyzed if infant self-comforting behaviors mediated the association between diagnostic group and child ToM-abilities. Regarding the Smarties-score, the total effect (*B* = 0.220, SE = 0.218, *t* = 1.007, *p* = 0.319, 95% CI = [−0.219; 0.658]), the direct effect (*B* = 0.248, SE = 0.217, *t* = 1.141, *p* = 0.259, 95% CI = [−0.188; 0.684]) as well as the indirect effect (*B* = −0.028, SE = 0.049, 95% CI = [−0.135; 0.068]) were not significant. The same accounted for the Maxi-score: the total effect (*B* = −0.036, SE = 0.209, *t* = −0.171, *p* = 0.865, 95% CI = [−0.456; 0.384]), the direct effect (*B* = −0.007, SE = 0.204, *t* = −0.036, *p* = 0.971, 95% CI = [−0.417; 0.402]) as well as the indirect effect (*B* = −0.028, SE = 0.060, 95% CI = [−0.147; 0.113]) were not significant.

## Discussion

Aim of this longitudinal study was to clarify the relation of self-regulatory abilities in infancy and later ToM in pre-school aged children of clinically depressed mothers and healthy controls.

The study highlights the role of infant’s self-comforting behavior for ToM development at pre-school age, as we found an interesting interaction effect indicating an association between self-comforting behavior at 3 months of age and child development of location belief understanding (Maxi-score) at pre-school age, specific for girls. Self-comforting behavior was assessed during interaction between the infant and its mother 3 months postpartum and therefore also includes information about dyadic interaction between the infant and its mother. As some studies reported increased self-comforting behaviors in infants of mothers with psychiatric disorders (Granat et al., 2017), increased self-comforting behaviors in infancy were seen as a necessary adaptation of the infant as their mothers lack sufficient dyadic affect regulation strategies. Therefore, increased self-comforting behaviors in infancy were seen as an indicator for an ineffective co-regulation of infant’s affect through the mother and therefore as an indicator for an insensitive parenting environment. The results of this study point in the direction that increased self-comforting behaviors (regardless of the conditions of origin) can be seen as an immense resource for child development and support functional affect and stress regulation strategies. Clearly, this association needs further investigation.

One explanation for the stronger association between infant’s self-comforting behavior and ToM in female children might be, that female infants are more sensitive to social signals, as it was shown that newborn and 12-month-old females show higher preferences for social stimuli than non-social stimuli in comparison to males (Connellan et al., 2000; Lutchmaya and Baron-Cohen, 2002). It is possible, that female infants are more sensitive to identify and adapt to insensitive maternal caregiving and that they are able to perpetuate the self-directed regulatory style into later development. This hypothesis is supported by a study of our work group, which revealed that female infants were more sensitive to low maternal bonding in the context of maternal anxiety disorders than male infants (Müller et al., 2016). Furthermore, child motoric development might account for this effect, as female infants develop mature motoric skills earlier in infancy than male infants (Dinkel and Snyder, 2020).

The relations between self-regulatory behaviors in infancy and later ToM which were observed in the present study were task specific (content vs. location false belief, understanding of own vs. other’s false belief). This finding is first evidence for a relation between self-regulation in infancy and later ToM. Clearly, it needs to be further explored. It appears that the present version of the Maxi task, which tested not only first order false belief understanding, but also tactical deception based on false belief understanding was a stricter test for ToM competence than the content false belief task, and was therefore more suitable for finding evidence for the assumed relationship. Further research needs to systematically address the generality of predictive relations of self-regulatory behavior in infancy and later ToM.

Contrary to our hypotheses, maternal postpartum or lifetime depression was not directly associated with child’s ToM at pre-school age. Although some studies showed a direct association between maternal depression and child’ ToM abilities (Rohrer et al., 2011; Shamblaw et al., 2019), other studies did not find such an association. A study by Greig and Howe (2001) only found differences in children’s emotion understanding, but not in their false belief competence. The results of this study add to the heterogeneous picture about the influence of maternal depression on children’ ToM and suggest that there must be other factors contributing to ToM development than maternal depression alone. Recent research indicates, that special characteristics of maternal depression account for the increased developmental risk of children of depressed mothers, such as chronicity, duration of the episode and severity (NICHD Early Child Care Research Network, 1999; Urizar and Muñoz, 2021). The study by Rohrer et al. (2011), which showed an association between maternal depression and ToM development in children, also reported that this association was especially significant if the mother suffered from recurrent depressive episodes. Furthermore, the timing of the depressive episode may exert influence on child development. As ToM develops during the first years of life, it can be assumed that child development is influenced by maternal mental disorder occurring during this time of the child’s life. Corresponding analyses should be targeted in future research.

There are some limitations to this study: due to a small sample size and subsequently reduced power, it was not possible to test for different clinical course patterns of maternal depression from postpartum period up to pre-school age. Furthermore, our sample was characterized by an over proportion of academic degrees.

Further research is needed to obtain specific baseline knowledge about the relationship between maternal depression and ToM development, taking self-comforting behavior into account. For example, the clinical course patterns of maternal depression from infancy up to pre-school age as well as the course and the origins of self-comforting behavior and their associations with ToM development are important research questions. Finally yet importantly, interactional behavior of the caregiver should also be addressed. Nevertheless, the results of this study highlight the influence of self-comforting behaviors in infancy for the development of ToM at pre-school age and shed further light on the discussion, how maternal depression influences child development.

## Data Availability Statement

The raw data supporting the conclusions of this article will be made available by the authors on request.

## Ethics Statement

The studies involving human participants were reviewed and approved by the Heidelberg University Medical Faculty. Written informed consent to participate in this study was provided by the participants and the participants’ legal guardian.

## Author Contributions

NN and MM equally contributed to analysis, and interpretation of the data, drafting of the manuscript, and approval of the final version. JT, A-LZ, and BS contributed to study and manuscript conception, interpretation of the results, and approval of the final version. CR contributed to study conception and design, drafting of the manuscript, and final approval of the final version. All authors contributed to the article and approved the submitted version.

## Conflict of Interest

The authors declare that the research was conducted in the absence of any commercial or financial relationships that could be construed as a potential conflict of interest.

## Publisher’s Note

All claims expressed in this article are solely those of the authors and do not necessarily represent those of their affiliated organizations, or those of the publisher, the editors and the reviewers. Any product that may be evaluated in this article, or claim that may be made by its manufacturer, is not guaranteed or endorsed by the publisher.
